# Sulfur Regulates the Trade-Off Between Growth and Andrographolide Accumulation *via* Nitrogen Metabolism in *Andrographis paniculata*

**DOI:** 10.3389/fpls.2021.687954

**Published:** 2021-07-14

**Authors:** Shao-Fen Jian, Xue-Jing Huang, Xiao-Nan Yang, Chu Zhong, Jian-Hua Miao

**Affiliations:** ^1^Guangxi Key Laboratory of Medicinal Resource Protection and Genetic Improvement, Guangxi Botanical Garden of Medicinal Plants, Nanning, China; ^2^Guangxi Engineering Research Centre of TCM Intelligent Creation, Guangxi Botanical Garden of Medicinal Plants, Nanning, China; ^3^College of Pharmacology, Guangxi Medical University, Nanning, China

**Keywords:** sulfur, nitrogen, *Andrographis paniculata* (Burm.f.) Nees, andrographolide accumulation, phytohormone

## Abstract

Nitrogen (N) and sulfur (S) are essential mineral nutrients for plant growth and metabolism. Here, we investigated their interaction in plant growth and andrographolide accumulation in medicinal plant *Andrographis paniculata* grown at different N (4 and 8 mmol·L^−1^) and S concentration levels (0.1 and 2.4 mmol L^−1^). We found that increasing the S application rate enhanced the accumulation of andrographolide compounds (AGCs) in *A. paniculata*. Simultaneously, salicylic acid (SA) and gibberellic acid 4 (GA_4_) concentrations were increased but trehalose/trehalose 6-phosphate (Tre/Tre6P) concentrations were decreased by high S, suggesting that they were involved in the S-mediated accumulation of AGCs. However, S affected plant growth differentially at different N levels. Metabolite analysis revealed that high S induced increases in the tricarboxylic acid (TCA) cycle and photorespiration under low N conditions, which promoted N assimilation and S metabolism, and simultaneously increased carbohydrate consumption and inhibited plant growth. In contrast, high S reduced N and S concentrations in plants and promoted plant growth under high N conditions. Taken together, the results indicated that increasing the S application rate is an effective strategy to improve AGC accumulation in *A. paniculata*. Nevertheless, the interaction of N and S affected the trade-off between plant growth and AGC accumulation, in which N metabolism plays a key role.

## Introduction

It is estimated that more than 80% of the worldwide population depends upon natural medicines for their healthcare (Choo et al., 2014). Most natural medicines are difficult to synthesize artificially or biologically, and they can only be obtained from medicinal plants. However, the growing need for plant-based medicines, health products, and pharmaceuticals, and the destructive exploitation of medicinal plants lead to increasing exhaustion of resources. Artificial cultivation of medicinal plants is an effective alternative way to solve this crisis.

During the “Green Revolution,” which started in the 1960s, the widespread use of nitrogen (N) chemical fertilizers in agricultural ecosystems ensured a sustained increase in cereal crop yield to meet the demand of the growing population (Funk et al., 2013). This has also had a profound impact on other agricultural production systems, such as the cultivation of medicinal plants. Although the high application rate of N fertilizer can obtain high biomass of plants, the contents of secondary bioactive ingredients of medicinal plants are usually reduced by increasing the N application rate (Liu et al., 2010a,b; Ibrahim et al., 2011; Wang et al., 2014; Medina-Pérez et al., 2015). Photosynthesis provides a source of assimilates for plant growth and metabolism. It has been generally recognized that a great part of photosynthate is used for growth and protein synthesis under sufficient and excessive N conditions, inhibiting the conversion of carbohydrates to C-rich secondary metabolites, such as phenols and terpenoids (Izmailov et al., 2018; Dong et al., 2019). Obviously, there is a trade-off between growth and secondary metabolism in plants, which could be mediated by N metabolism (Hakulinen et al., 1995; Su et al., 2010). To uncover the underlying mechanism that N involves in the regulation of the trade-off between plant growth and secondary metabolism is of great significance to improve the yield and quality of cultivated medicinal plants.

Sulfur (S) is a macronutrient that could not be neglected and is critical for plant growth and formation of yield and quality of fruits, vegetables, tobacco leaves, etc. (Blue et al., 1981; Malhi et al., 2007; Kok et al., 2011; Meena et al., 2015). Inorganic S is assimilated into cysteine (Cys) by a set of reactions and subsequently into methionine (Met) and secondary S compounds, such as biotin, glucosinolates, and thiol compounds, which provide the characteristic taste and smell of foods, and many of which play important roles in health promotion and protective properties (Fismes et al., 1997; Sexton et al., 2002; Droux, 2004; Mugford et al., 2011; Kopriva et al., 2016). Recent studies have revealed that S affected the accumulation of indigotine and indirubin, major active ingredients in the medicinal plant *Isatis indigotica* (Miao et al., 2019). However, little is known about how S regulates secondary metabolism in medicinal plants.

There are significant interactions between N and S on the growth, nutritional components, and secondary metabolites of plants (Paek et al., 2000; Fernando and Miralles, 2007; Miao et al., 2018). S and N showed reciprocal influences on the assimilation of each other in plants (Carfagna et al., 2011; Xu et al., 2017). Lack of root nitrate reductase (NR) decreased S uptake rate in N-grown tobacco plants, as well as root S and total S contents irrespective of N nutrition (Kruse et al., 2008). S is also a necessary element for N metabolism (Friedrich and Schrader, 1978; Ingenbleek and Young, 2004). S increased the activities of enzymes in N assimilation and transamination to promote N metabolism (Li et al., 2013). S deprivation affected early N_2_ fixation and subsequent C and N metabolism in lucerne (Deboer and Duke, 2010). The effect of S nutrition on C metabolism also seems to be related to that on N metabolism, e.g., production of certain proteins and enzymes (Qi, 1989).

*Andrographis paniculata* (Burm.f.) Nees (known as “Chuanxinlian” in China) is a member of the Acanthaceae family that is widely used as an anti-inflammatory and antipyretic drug for the treatment of cold, fever, laryngitis, and diarrhea in traditional medicinal systems of Indian, Chinese, Thai, etc. (Tang and Eisenbrand, 1992; Subramanian et al., 2012). Its major bioactive constituents, andrographolide compounds, (AGCs; Pholphana et al., 2013), which belong to diterpene lactones, are also stated as anticancer, antibacterial, antivirus and anti-hepatitis drugs (Aromdee, 2012; Xu et al., 2012; Talei et al., 2014; Valdiani et al., 2014). Since it is difficult to be synthesized artificially, it is mainly obtained from plant materials. To enhance the content of AGCs is therefore a major purpose of cultivation and breeding of this plant species (Valdiani et al., 2014; Verma et al., 2015). It has been proposed that the agronomical characteristics, such as single plant leaf and branching numbers, and biomass of *A. paniculata* plants were negatively correlated with contents of AGCs (Zeng et al., 2019), suggesting the trade-off between plant growth and AGC production in *A. paniculata*. Nevertheless, their interrelationship remains to be elucidated.

Given that S is an important quality-determining nutrient element of crops and closely related to N metabolism, we speculated that S could coordinate plant growth and AGC accumulation in *A. paniculata* through its interaction with N metabolism. In this study, we aimed to investigate the interaction of S and N on plant growth and AGC accumulation in *A. paniculata*, and reveal the physiological mechanism that S coordinates plant growth and secondary metabolism. The results are of great significance to obtain high yield and quality of *A. paniculata* by improving its nutrient management.

## Materials and Methods

### Plant Growth Condition and Treatment

A factorial (2 N × 2S treatments) pot experiment was conducted in a glasshouse at Guangxi Botanical Garden of Medicinal Plants, Nanning, China (108°22′E, 22°51′N). *A. paniculata* seeds were sown on a seedbed for germination and growth. Seedlings with five pairs of true leaves were transplanted to pots containing 5 L of perlite. There were four plants in each pot. All the seedlings were divided into four groups (treatments) and supplied by nutrient solution with the combination of different concentration levels of −N (4 and 8 mmol L^−1^) and S (0.1 and 2.4 mmol L^−1^). The mineral nutrition composition of the nutrient solution in each treatment is listed in Supplementary Table S1. The pH of the solution was adjusted to 6. In the first week after transplanting, only deionized water was supplied to the seedlings to revive them and deplete inorganic N in the plants. Then, the seedlings were supplied with a 200-ml nutrient solution per pot every 3 or 4 days.

After 2 months of treatment, the plants were sampled for physiological and biochemical analysis.

### Photosynthesis Measurements

The photosynthetic gas exchange in fully expanded new leaves on the main stem was measured using a portable photosynthesis system (LI-6400*XT*, LI-COR, Lincoln, NE, United States) with an open-flow gas exchange system. The photosynthetic photon flux density (PPFD) in the leaf chamber was 800 μmol m^−2^ s^−1^, and the temperature was 25°C. The leaves were put in the chamber to adapt for 10 min. When the data reached a steady-state, net photosynthetic rate (*P*_n_, μmol m^−2^ s^−1^), stomatal conductance (*g*_s_, mol m^−2^ s^−1^), intercellular CO_2_ concentration (*C*_i_, μmol mol^−1^), and transpiration rate (*T*_r_, μmol m^−2^ s^−1^) were recorded.

### Measurements of Plant Growth Traits and Total Concentrations of Nitrogen and Sulfur

The plant height of four individuals in each treatment was measured. Then, the plants were separated into leaves, stems, and roots. Leaf area was measured using a plant image processing system (model LA-S, Wanshen Ltd., Hangzhou, China). The samples were then oven-dried at 60°C for 72 h and pulverized after getting weighed.

To measure total N in the leaves, pulverized dry leaf samples (about 100 mg) were digested with H_2_SO_4_-H_2_O_2_ at 260°C, and total N in the solution was quantified with an Auto-Kjeldahl apparatus (model K1100, Hanon, China). Another 200 mg samples were digested with HNO_3_-H_2_O_2_ in a boiling water bath to determine the concentration of S by the inductively coupled plasma mass spectrometry (ICP-MS) method (NexION 350X, PerkinElmer, Waltham, MA, United States). The acidity of the solution for ICP-MS determination was controlled lower than 2%.

### Metabolome Profiling

Fully expanded new leaves were sampled, frozen in liquid nitrogen immediately, and stored at −80°C for biochemical measurements.

Leaf samples (100 mg) were individually ground with liquid nitrogen, and the powder was re-suspended with pre-chilled 80% methanol and 0.1% formic acid by well vortexing. The samples were incubated at 4°C for 5 min and then centrifuged at 15,000 × *g* and 4°C for 5 min. Some of the supernatants were diluted to a final concentration containing 60% methanol with LC-MS grade water. The samples were subsequently transferred to a fresh Eppendorf tube with a 0.22-μm filter and then centrifuged at 15,000 × *g* and 4°C for 10 min. Finally, the filtrate was injected into the liquid chromatography with a tandem mass spectrometry (LC-MS/MS) system for analysis.

LC-MS/MS analyses were performed using a Vanquish UHPLC system (Thermo-Fisher, Waltham, MA, United States) coupled with an Orbitrap Q Exactive series mass spectrometer (Thermo-Fisher, Waltham, MA, United States). The samples were injected onto a Hypersil Gold column (100 × 2.1 mm, 1.9 μm, Thermo-Fisher, Waltham, MA, United States) using a 16-min linear gradient at a flow rate of 0.2 ml min^−1^. The eluents for the positive polarity mode were 0.1% formic acid in water (A) and methanol (B). The eluents for the negative polarity mode were 5 mmol L^−1^ ammonium acetate, pH 9 (A) and methanol (B). The solvent gradient was set as follows: 2% B, 1.5 min; 2–100% B, 12 min; 100% B, 14 min; 100–2% B, 14.1 min; 2% B, 16 min. Q executive mass series spectrometer was operated in positive/negative polarity mode with a spray voltage of 3.2 kV, capillary temperature of 320°C, sheath gas flow rate of 35 arb, and aux gas flow rate of 10 arb.

### Nitrogen Metabolic Enzyme Assays

Nitrate reductase (NR) in frozen leaf samples was extracted by homogenizing with 25 mmol L^−1^ potassium phosphate buffer (pH 7.5) containing 10 mmol L^−1^ l-cysteine and 1 mmol L^−1^ EDTA-Na_2_, followed by centrifuging at 10,000 × *g* and 4°C for 10 min. The NR activity in the supernatant was measured colorimetrically at 540 nm as described in Hageman and Reed (1980) by monitoring the generation rate of NO_2_^−^.

Another 100 mg samples were homogenized with an extracting agent containing 50 mmol L^−1^ Tris-HCl (pH 8), 2 mmol L^−1^ Mg^2+^, 2 mmol L^−1^ DTT, and 0.4 mol L^−1^ sucrose. The homogenates were centrifuged at 10,000 × *g* and 4°C for 10 min, and the supernatants were used for subsequent measurements. The activity of glutamine synthetase (GS) was measured according to the method described in Loyola-Vargas and de Jimenez (1984), and the activities of glutamic-oxaloacetic transaminase (GOT) and glutamic-pyruvic transaminase (GPT) were measured using the method described in Wu et al. (1998).

### Determination of Andrographolide and Dehydroandrographolide

About 100 mg pulverized dry leaf samples were immersed in 25 ml 50% methanol for 1 h and treated ultrasonically for 30 min followed by filtering. The loss weight of extraction during that process was supplemented with 50% methanol. Extracted andrographolide and dehydroandrographolide were measured using the ultra performance LC-MS method (Cheng et al., 2014). The mobile phase was implemented with methanol-water (48:52 v/v) at a flow rate of 1 ml s^−1^. The column temperature and sample sizes were 30°C and 10 μl, respectively. Andrographolide and dehydroandrographolide were detected at 225 nm and 254 nm, respectively. The concentrations of andrographolide and dehydroandrographolide in the leaf samples were calculated from the standard curves made by the linear relationship between peak areas and standard concentration of andrographolide/dehydroandrographolide.

### Data Processing and Statistical Analysis

Two-way ANOVAs were performed to analyze the effects of N and S, and their interactions on the growth and physiological and biochemical indexes. Taking the combination of N and S as an independent variable, multiple comparisons were performed using the method least significant difference (LSD) test. Differences were considered statistically significant when *p* < 0.05.

The raw metabolome data files generated by UHPLC-MS/MS were processed using the Compound Discoverer 3.1 (CD3.1, Thermo-Fisher, Waltham, MA, United States) to perform peak alignment, peak picking, and quantitation for each metabolite. The main parameters were set as follows: retention time tolerance, 0.2 min; actual mass tolerance, 5 ppm; signal intensity tolerance, 30%; signal/noise ratio, 3; and minimum intensity, 100,000. After that, peak intensities were normalized to the total spectral intensity. The normalized data were used to predict the molecular formula based on additive ions, molecular ion peaks, and fragment ions. Then, the peaks were matched with the mzCloud^1^ and ChemSpider^2^ databases to obtain accurate qualitative and relative quantitative results. Statistical analyses were performed using the statistical software R (R version R-3.4.3), Python (Python 2.7.6 version), and CentOS (CentOS release 6.6). When the data were not normally distributed, normal transformations were attempted using the area normalization method. A blank sample was used for normalization of the data.

## Results

### Plant Agronomical Characteristics and Photosynthesis

The plants grown with low N (4 mmol L^−1^) displayed a lighter leaf color than those grown with high N (8 mmol L^−1^), and accumulated anthocyanin in the bottom leaves (Supplementary Figure S1a). It is speculated that the plants grown with 4 mmol L^−1^ N suffered low N stress to a certain extent.

**Figure 1:**
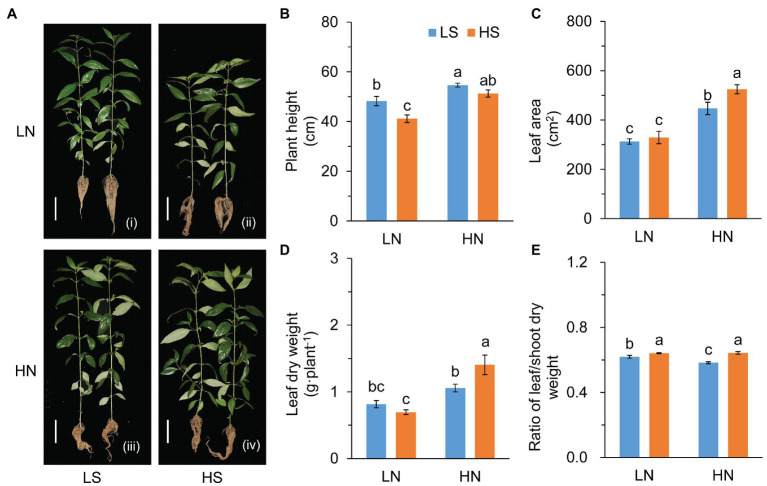
Growth evaluation of *A. paniculata* plants grown under different nitrogen and sulfur conditions. **(A)** Plants treated with different N and S levels were pictured after harvesting. **(B)** Plant height. **(C)** Leaf area measured by photographing. **(D)** Leaf dry weight. **(E)** The ratio of leaf/shoot dry weight. Data were presented as mean ± SE (*n* = 4). Different letters on the bars indicate significant difference among treatments at *p* < 0.05 using the method of *LSD*. Scar bars = 10 cm. LN, low nitrogen; HN, high nitrogen; LS, low sulfur; HS, high sulfur.

S affected plant growth remarkably (Figure 1). In comparison to low S, high S reduced plant height under low N conditions, but increased the leaf area under high N conditions (Figures 1B,C). The increase in the leaf area was possibly due to the growth of branches (Figure 1A) rather than an increase in leaf number on the main stem (Supplementary Figure S1a). High S slightly reduced leaf dry weight under low N conditions but remarkably increased leaf dry weight under high N conditions (Figure 1D). The ratio of leaf/shoot dry weight was significantly increased with higGrowthh S under both N conditions (Figure 1E).

Photosynthetic rate (*P*_n_) was inhibited by high S under low N conditions, but it was not affected under high N conditions (Supplementary Figure S1b). The decrease in *P*_n_ was probably due to stomatal closure (Supplementary Figures S1c–e). The results indicated that the effects of S on the growth of *A. paniculata* varied with N levels.

### Accumulation of Andrographolide Compounds, Nitrogen, and Sulfur

**Figure 2:**
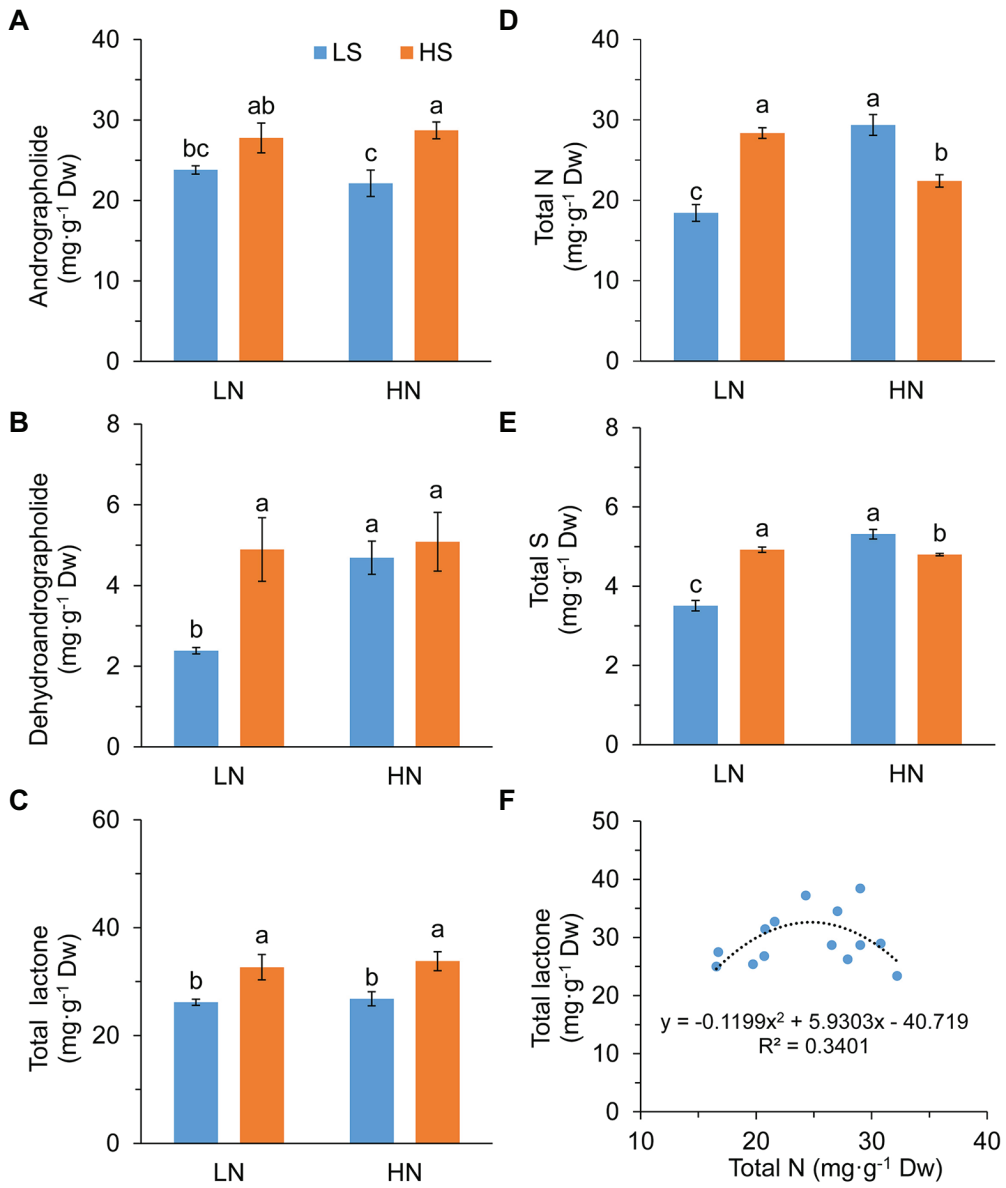
Diterpene lactones, nitrogen, and sulfur concentration and the relationship between N and lactone in leaves of *A. paniculata* plants grown under different nitrogen and sulfur conditions. **(A)** Andrographolide concentration. **(B)** Dehydroandrographolide concentration. **(C)** Total lactone (andrographolide + dehydroandrographolide) concentration. **(D)** Total leaf N concentration. **(E)** Total leaf S concentration. **(F)** The relationship between total leaf N concentration and total lactone concentration. Data were presented as mean ± SE (*n* = 4). Different letters on the bars indicate significant difference among treatments at *p* < 0.05 using the method of *LSD*. LN, low nitrogen; HN, high nitrogen; LS, low sulfur; HS, high sulfur.

High S increased leaf andrographolide and dehydroanddrographolide concentrations with high N and low N, respectively (Figures 2A,B). As a result, the concentration of total diterpene lactones (andrographolide + dehydroanddrographolide) was significantly increased with high S at both N levels (Figure 2C).

Leaf N concentration was in parallel with S concentration in response to high S that they were increased at low N but decreased at high N (Figures 2D,E). However, total amounts of leaf N and S expressed on a “per plant” basis (mg·plant^−1^) were not significantly affected by high S under both N conditions (Supplementary Figure S2). There was a parabolic relationship between the concentrations of leaf N and total diterpene lactones (*F* = 2.834, *p* = 0.102; Figure 2F). The highest concentration of total diterpene lactones was obtained when leaf N was about 25 mg g^−1^ Dw.

These results suggested that increasing the S application rate enhanced the accumulation of diteroene lactones in *A. paniculata*, which was associated with leaf N accumulation.

### Activities of Nitrogen Metabolic Enzymes

**Figure 3:**
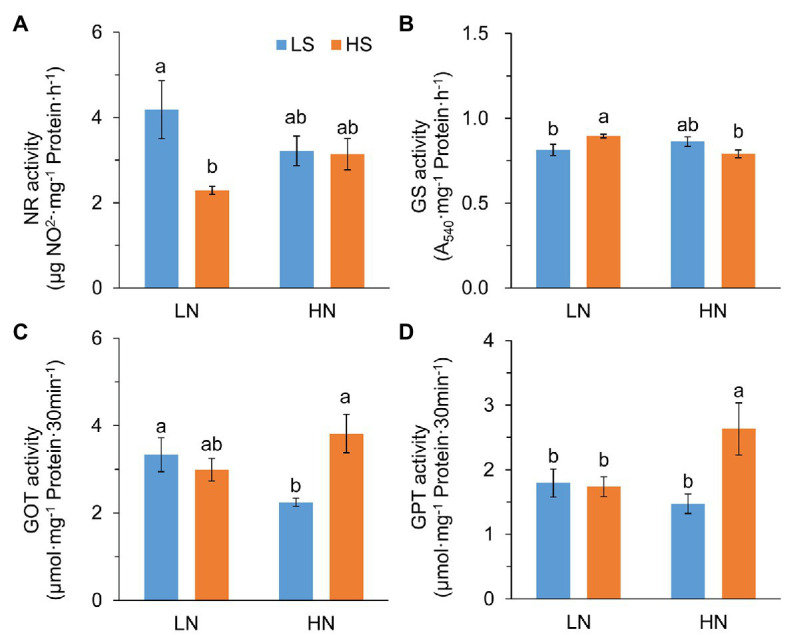
Activity of N metabolic enzymes in leaves of *A. paniculata* plants grown under different nitrogen and sulfur conditions. **(A)** Nitrate reductase. **(B)** Glutamine synthase. **(C)** Glutamic-oxalacetic transaminase. **(D)** Glutamic-pyruvic transaminase. Data were presented as mean ± SE (*n* = 4). Different letters on the bars indicate significant difference among treatments at *p* < 0.05 using the method of *LSD*. LN, low nitrogen; HN, high nitrogen; LS, low sulfur; HS, high sulfur.

To verify whether S affected N metabolism, we analyzed the activities of N metabolic enzymes. Nitrate reductase (NR) activity was significantly reduced with high S at low N, but it was not affected at high N (Figure 3A). Glutamine synthase (GS) activity was remarkably increased with high S at low N but slightly decreased at high N (Figure 3B). Glutamic-oxaloacetic transaminase (GOT) and glutamic-pyruvic transaminase (GPT) activities were not affected by S at low N, while they were significantly increased by high S at high N (Figures 3C,D). The results indicated that S affected N metabolism differentially under the two N conditions.

### Carbon and Nitrogen Metabolites

**Figure 4:**
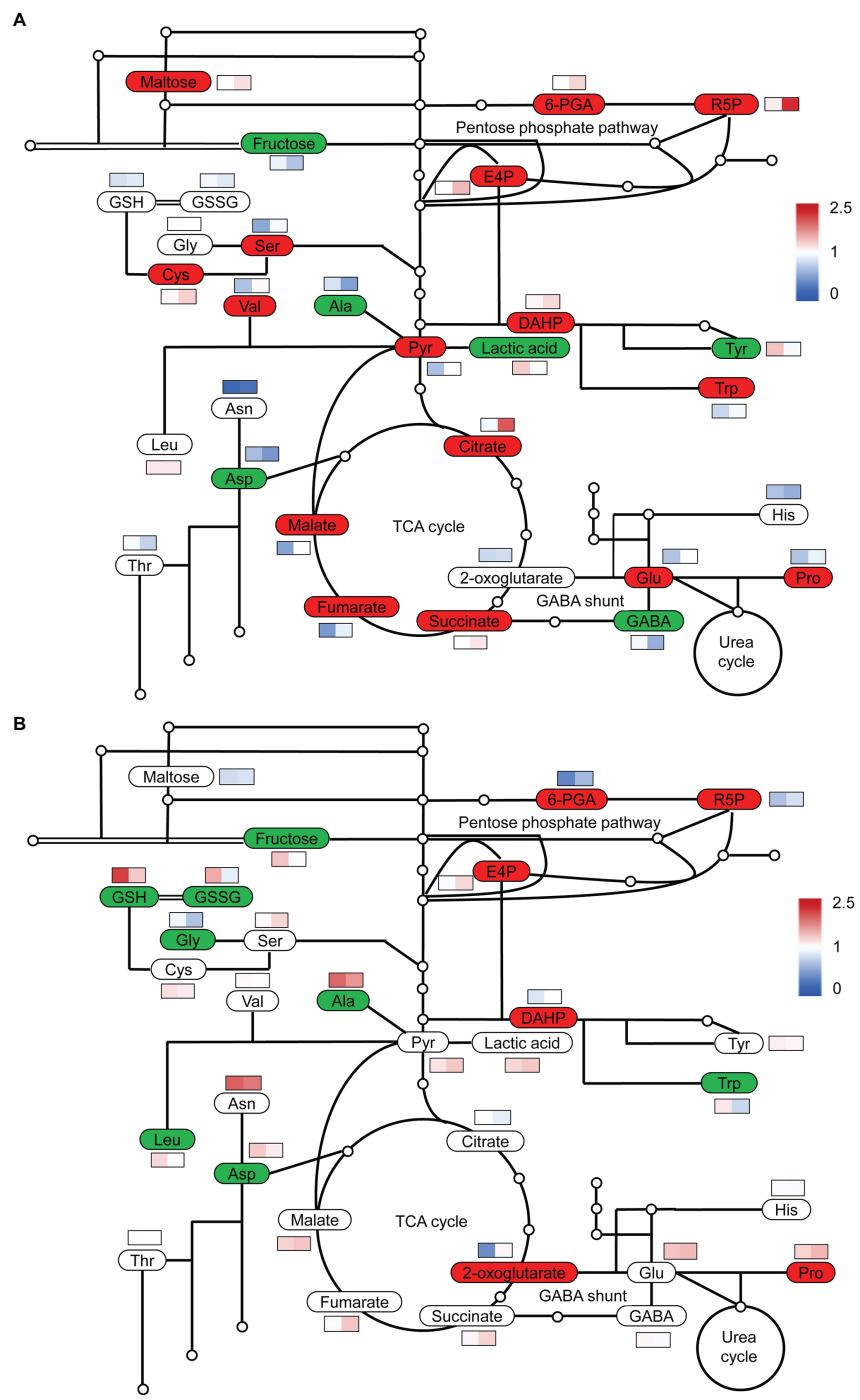
Simplified diagram of changes in carbon and nitrogen metabolites in leaves of *A. paniculata* plants grown under different nitrogen and sulfur conditions. **(A)** Changes in metabolites in high S compared with low S under low N conditions. **(B)** Changes in metabolites in high S compared with low S under high N conditions. Red represents significantly increased, green represents significantly decreased, and white represent not significant. Heat map in the rectangle meant relative values under low S and high S conditions on the left and right, respectively. Data were presented as means obtained from four biological replicates.

To obtain an overview of the effects of S on C and N metabolism, we detected the changes in some carbohydrates, organic acids, and amino acids. At low N, high S increased the contents of pyruvate and organic acids in the TCA cycle, such as citrate, malate, fumarate, and succinate in comparison with low S, but the 2-oxoglutarate (2-OG) level was not affected (Figure 4A). The levels of Ser, Cys, Val, Glu, Pro, and Trp were considerably increased with high S, while the levels of GABA, Asp, Ala, and Tyr were remarkably reduced.

At high N, pyruvate and organic acids in the TCA cycle were not affected with high S with the exception of 2-OG, which was remarkably accumulated with high S (Figure 4B). The levels of Ala, Asp, Gly, Leu, and Trp were decreased with high S, but only the Pro concentration was increased. The levels of GSSG and GSH were also remarkably decreased. However, both GSSG and GSH were higher in high N than in low N.

Under both N conditions, fructose was decreased, but the pentose phosphate pathway (PPP) was enhanced, resulting in an increased supply of erythritose 4-phosphate (E4P) for the synthesis of aromatic amino acids as indicated by the increase in 3-deoxy-D-arabinoheptanoic acid-7-phosphate (DAHP; Figures 4A,B), which is generated from E4P and phosphoenolpyruvate (PEP). Maltose was remarkably increased with high S under low N conditions, but it was not affected under high N conditions. E4P and DAHP were greater under low N conditions than under high N conditions.

**Figure 5:**
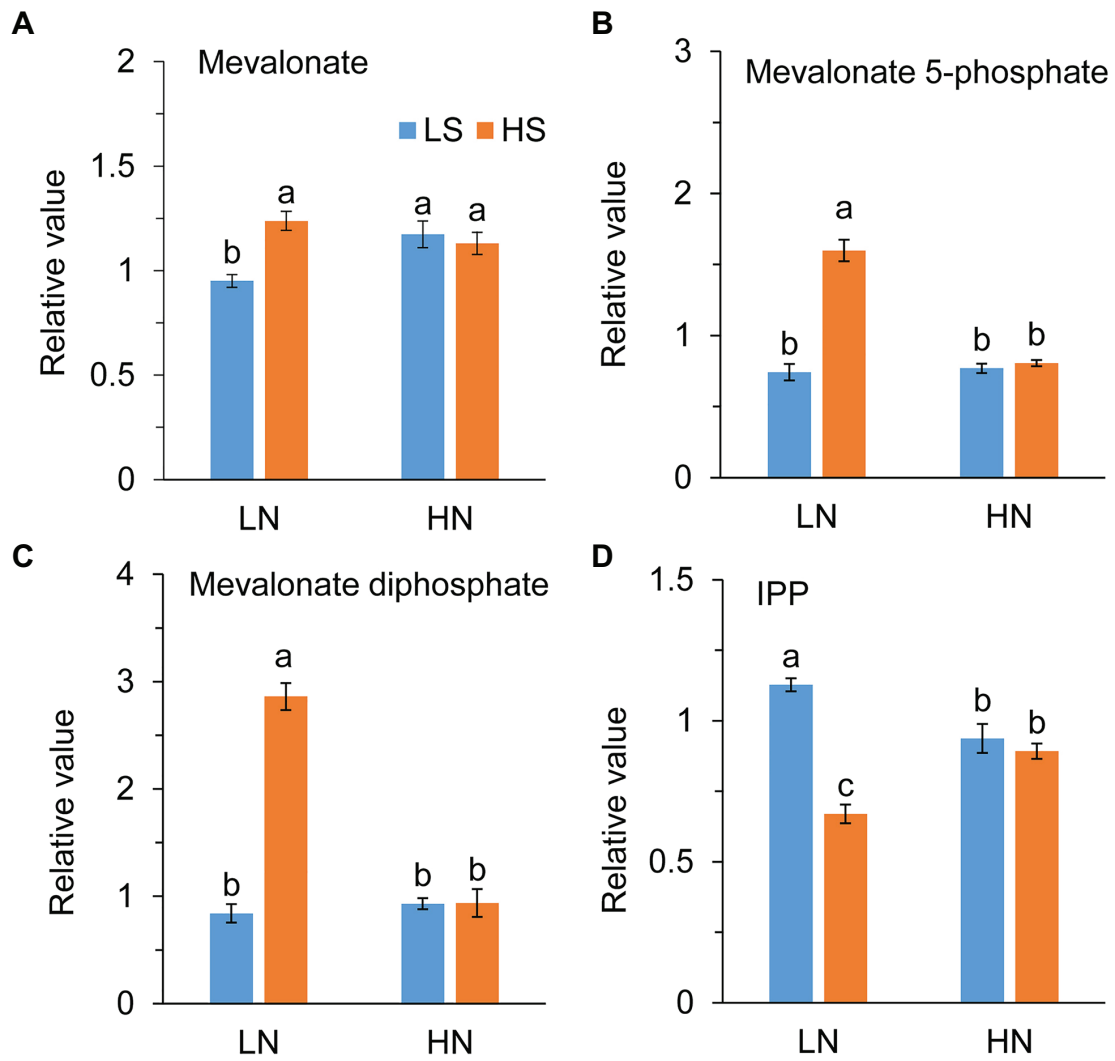
Intermetabolites of terpene metabolism in leaves of *A. paniculata* plants grown under different nitrogen and sulfur conditions. **(A)** A Relative value of mevalonate. **(B)** Relative value of mevalonate 5-phosphate. **(C)** Relative value of mevalonate diphosphate. **(D)** Relative value of isopentenyl pyrophosphate (IPP). Data were presented as mean ± SE (*n* = 4). Different letters on the bars indicate significant difference among treatments at *p* < 0.05 using the method of *LSD*. LN, low nitrogen; HN, high nitrogen; LS, low sulfur; HS, high sulfur.

Acetyl-CoA is the initial precursors of IPP synthesized from the MVA pathway. Consistent with the enhancement of the TCA cycle, high S increased the levels of mevalonate, mevalonate 5-phosphate, and mevalonate biphosphate at low N, as shown in Figures 5A–C. However, IPP, the precursor of terpenoids, was remarkably reduced with high S (Figure 5D). At high N, those metabolites were not different between low S and high S.

### Phytohormones and Trehalose Metabolism

**Figure 6:**
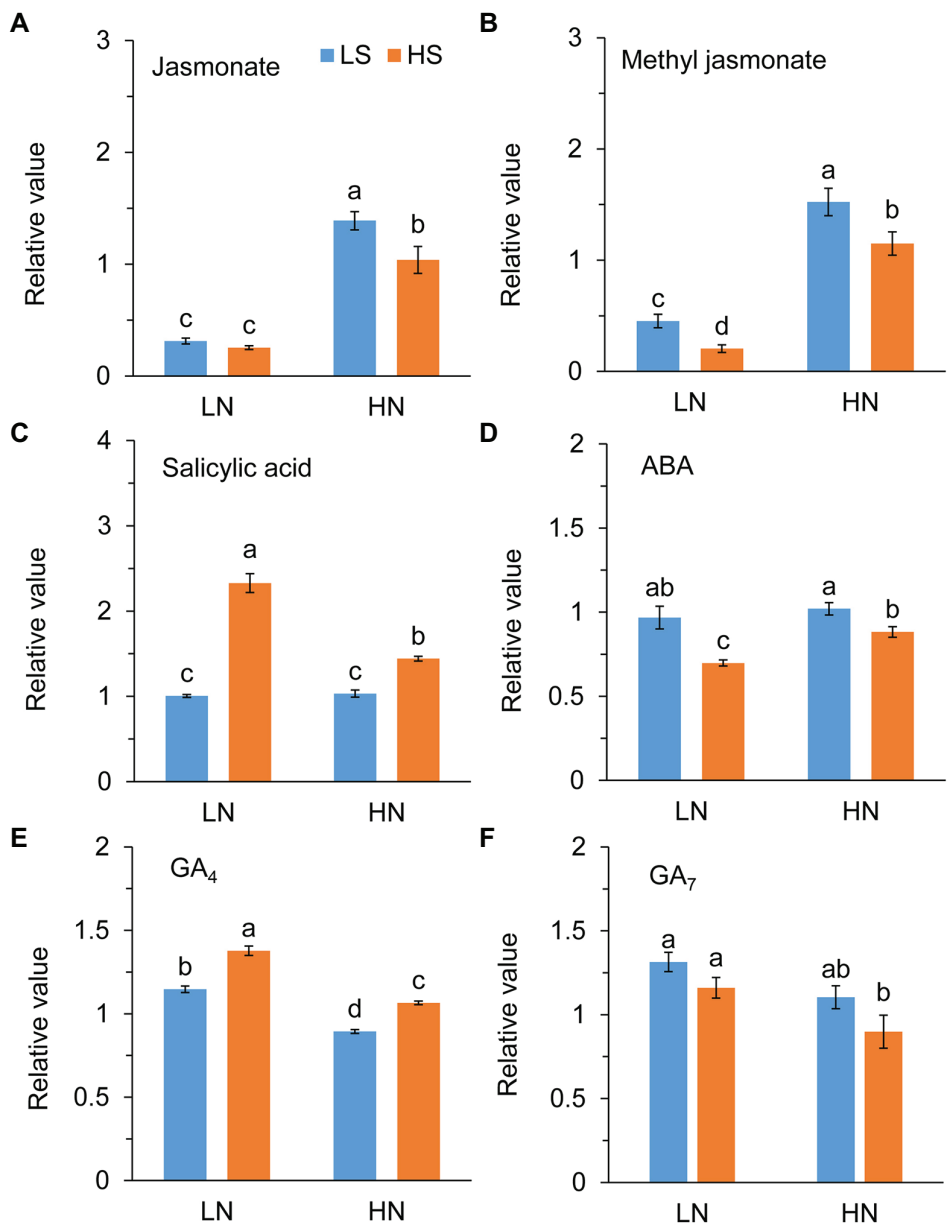
Phytohormones in leaves of *A. paniculata* plants grown under different nitrogen and sulfur conditions. **(A)** Relative value of jasmonate. **(B)** Relative value of methyl jasmonate. **(C)** Relative value of salicylic acid. **(D)** Relative value of abscisic acid (ABA). **(E)** Relative value of gibberellic acid 4 (GA_4_). **(F)** Relative value of gibberellic acid 7 (GA_7_). Data were presented as mean ± SE (*n* = 4). Different letters on the bars indicate significant difference among treatments at *p* < 0.05 using the method of *LSD*. LN, low nitrogen; HN, high nitrogen; LS, low sulfur; HS, high sulfur.

**Figure 7:**
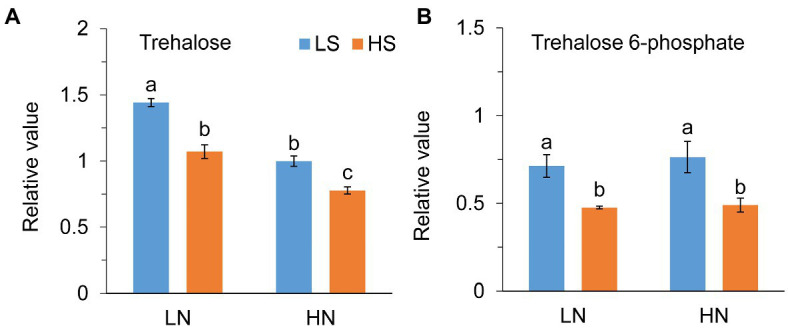
Trehalose **(A)** and trehalose 6-phosphate **(B)** levels in leaves of *A. paniculata* plants grown under different nitrogen and sulfur conditions. Data were presented as mean ± SE (*n* = 4). Different letters on the bars indicate significant difference among treatments at *p* < 0.05 using the method of *LSD*. LN, low nitrogen; HN, high nitrogen; LS, low sulfur; HS, high sulfur.

Jasmonate (JA) level was not different between low S and high S under low N conditions, but it was remarkably reduced with high S under high N conditions (Figure 6A). Methyl jasmonate (MeJA) and ABA levels were reduced with high S under both N conditions (Figures 6B,D). In contrast, high S significantly increased salicylic acid (SA) and GA_4_ levels under both N conditions (Figures 6C,E), while GA_7_ was not affected (Figure 6F). Trehalose (Tre) and trehalose 6-phosphate (Tre6P) levels were decreased with high S under both N conditions (Figure 7).

## Discussion

### Interaction of Nitrogen and Sulfur on Plant Growth and Andrographolide Compound Accumulation

S, like its counterpart C and N, plays a pivotal role in plant development and metabolism. It has been reported that S promotes plant growth and improves the quality of products, and these positive effects were associated with the conditions of high N application (Xie et al., 2003; Kong et al., 2013). In agreement with these findings, the results revealed that there was a significant interaction between S and N on the growth and AG accumulation of *A. paniculata* plants (Figures 1, 2). Increased S application rate displayed opposite effects on plant growth under the two N conditions. The productivity of photosynthesis could explain the different effects of S on the growth of *A. paniculata* plants under different N conditions (Supplementary Figure S1b). However, high S increased the accumulation of AGCs under both N conditions. The results suggested that S is a key factor mediating the trade-off between plant growth and AG biosynthesis in *A. paniculata* plants.

N plays versatile roles in regulating secondary metabolism in medicinal plants. It has been considered to function as a competitor of C-skeleton or implicated as a signal in the biosynthesis of secondary metabolites (Fritz et al., 2006; Izmailov et al., 2018). A number of studies have proposed that increasing the N application rate reduces the accumulation of C-rich secondary metabolites, such as terpenoid and flavanoid (Liu et al., 2010a,b; Medina-Pérez et al., 2015), which conforms to the carbon/nutrition balance hypothesis (CNBH; Bryant et al., 1983). However, some studies, those that do not support this view, have suggested that N promotes the biosynthesis of secondary metabolites as the component of key enzymes in secondary metabolism pathways (Hamilton et al., 2001). The results in this study showed that the concentration of total diterpene lactones increased linearly with leaf N concentration under low N conditions (*F* = 7.439, *p* = 0.041, R^2^ = 0.598) and decreased linearly with leaf N concentration under high N conditions (*F* = 5.457, *p* = 0.067, R^2^ = 0.522). The highest value was obtained when leaf N was about 25 mg·g^−1^ (Figure 2F). It is implied that the biosynthesis of AGCs was mainly limited with enzyme abundance under low leaf N concentration, while it was constrained under high leaf N concentration because of increased competition of the C-skeleton by N assimilation. Under both N conditions, high S increased the concentration of AGCs with the corresponding leaf N concentration in an appropriate range, indicating that S could regulate AGC biosynthesis *via* N metabolism.

It has been reported that S application increased plant N absorption because of the improvement in N assimilation (Fazili et al., 2008). We found that N assimilation was affected quite differentially by S at different N levels in this study. Under low N conditions, high S decreased NR activity but increased GS activity, while GS activity was decreased under high N conditions (Figures 3A,B). These results were well in line with the concentration of leaf N, suggesting that increasing the S application rate affected plant N uptake and primary assimilation. Nevertheless, it is confusing that leaf N concentration in high N was significantly lower than that in low N under high S conditions. It most probably resulted from the dilution effect caused by increased leaf dry weight, because high N increased the total amount of leaf N by 53.4% but increased leaf dry weight two times in comparison to low N. Similarly, the increased leaf dry weight had a dilution effect on S as well.

### Sulfur-Regulated Trade-Off Between Growth and Andrographolide Compound Accumulation Was Associated With the Balance in Carbon and Nitrogen Metabolism

N nutrient alters plant primary and secondary metabolisms and, therefore, regulates the trade-off between growth and defense responses (Bot et al., 2009). 2-oxoglutarate (2-OG) provides C-skeleton for N assimilation. The variation in N assimilation was closely related to the change in organic acids in the TCA cycle (Zhong et al., 2019). GABA acts as an important C and N source, which maintains the TCA cycle especially under N starvation conditions (Solomon and Oliver, 2002). In this study, high S induced the accumulation of organic acids with the exception of 2-OG in the TCA cycle under low N conditions. Accordingly, Glu was considerably accumulated, and the GABA shunt was increased. In contrast, high S led to the accumulation of 2-OG at high N, while other organic acids in the TCA cycle and Glu were not significantly changed (Figure 4). These results also supported the notion that S increased N assimilation under low N conditions. The previous study revealed that reducing N assimilation synergistically promoted plant growth and andrographolide biosynthesis in *A. paniculata* (Zhong et al., 2021). Enhanced N assimilation could increase the consumption of carbohydrates, which is not conducive to the accumulation of biomass.

On the other hand, acetyl-CoA provides a precursor for terpene biosynthesis *via* the MVA pathway. Enhanced TCA cycle in the conditions of low N combined with high S promoted AGC biosynthesis, which was indicated by the increases in mevalonate, mevalonate-5-phosphate, and mevalonate-diphosphate, and decrease in IPP (Figure 5) in the MVA pathway. Under high N conditions, these intermediate metabolites in the MVA pathway were not affected by high S, although the AGC concentration was increased. It is speculated that the AGCs of plants grown under high N conditions could be synthesized through the MEP pathway, because both the MVA and MEP pathways are involved in AGC biosynthesis, and MEP is the major biosynthetic pathway to these diterpenoids (Srivastava and Akhila, 2010).

S and N are important constituent elements of amino acids. High S showed significant effects on total S and sulfate contents (Figure 2E; Supplementary Figure S3a), which was consistent with that of total N, suggesting that S and N were intimately connected. Ser, Cys, Met, and Val are S metabolism-related amino acids. Ser is a marker amino acid in the photorespiration pathway, which is involved in the synthesis of Cys and Met, followed by the S-adenosyl-l-homocysteine (SAH)/S-adenosyl-methionine (SAM) cycle (Abadie and Tcherkez, 2019). Here, we observed significant increases in Ser, Cys, Val (Figure 4A), and SAH (Supplementary Figure S3b) and a decrease in SAM (Supplementary Figure S3c) under the conditions of high S combined with low N rather than high N. Photorespiration stimulates plant N and S metabolism (Bloom, 2015; Abadie and Tcherkez, 2019; Wujeska-Klause et al., 2019), and plays an important role in plant adaptation to low N conditions (Tang et al., 2018). It is probably that high S-stimulated photorespiration improved S and N metabolism and finally increased their accumulation under low N conditions. Another support for this view came from the increased activity of GS, whose chloroplastic GS2 isoform is responsible for the re-assimilation of NH_4_^+^ derived from photorespiration (Thomsen et al., 2014; Zhong et al., 2019). On the other hand, photorespiration increased the consumption of carbohydrates and consequently reduced the photosynthesis and growth of plants (Kang et al., 2018).

Additionally, high S increased maltose concentration and maintained relatively higher levels of 6-phosphogluconic acid (6PGA) and ribulose 5-phosphate (R5P) in plants grown with low N in comparison to those in high N, indicating that high S increased sugar catabolism under low N conditions. The partitioning of S between glutathione and protein synthesis determines plant growth (Speiser et al., 2018). The GSSG and GSH levels in plants grown under high N conditions were remarkably decreased with high S, which could contribute to better growth under those conditions.

Taken together, the results revealed that the balance between C and N metabolism is critical in regulating the trade-off between growth and AGC accumulation in *A. paniculata*. S-induced increase in N metabolism associated with enhanced consumption of carbohydrates *via* the TCA cycle and photorespiration were the main reasons for the discordant between growth and AGC biosynthesis under low N conditions. Contrary to low N conditions, high S increased the transamination of amino acids rather than N assimilation and photorespiration under high N conditions. However, the relationship between N metabolism and secondary metabolism still needs to be clarified.

### Signal Molecules Involved in Sulfur-Regulated Andrographolide Biosynthesis

A complex signaling network integrating phytohormones and metabolites regulates the biosynthesis of secondary metabolites in plants (Wang and Wu, 2005; Karppinen et al., 2016). Jasmonate (JA), abscisic acid (ABA), salicylic acid (SA), and gibberellic acids (GAs) are important plant hormones involved in the biosynthesis and accumulation of bioactive secondary metabolites in medicinal plants (Gao et al., 2012; Liang et al., 2013; Afrin et al., 2015; Yang et al., 2020). The exogenous application of these stimuli regulated the expression of genes/proteins in the biosynthetic pathways of secondary metabolites (Lee et al., 2008; Kiselev et al., 2010; Yang et al., 2020). It has been reported that MeJA induced andrographolide biosynthesis by the upregulation of genes in the MVA and MEP pathways (Sharma et al., 2015; Gao et al., 2019; Sun et al., 2019). However, we found in this study that high S application decreased the endogenous levels of JA, MeJA, and ABA but increased the SA and GA_4_ levels in the *A. paniculata* leaves, suggesting that SA and GA_4_ rather than ABA and JA/MeJA could be involved in S-mediated AGC biosynthesis.

Tajidin et al. (2019) compared the metabolome in young and mature leaves of *A. paniculata* and found that increase in carbohydrate accumulation was accompanied by a decrease in andrographolide in mature leaves compared with young leaves. Their results suggested that carbohydrate accumulation caused by leaf senescence impeded andrographolide biosynthesis. Trehalose (Tre) and trehalose 6-phosphate (Tre6P) are important signal metabolites linking plant growth and development and metabolism to C status in plants (Paul, 2008; O’Hara et al., 2013; Figueroa and Lunn, 2016). The previous study has revealed that Tre6P was responsive to changes in N status but not to changes in P and S status in plants (Yadav et al., 2014). In this study, however, high S decreased both Tre and Tre6P at different N levels. The changes in Tre and Tre6P reflect the fluctuation of sucrose in the plant; and, in general, their high accumulation eventually results in plant senescence (Schluepmann et al., 2004; Wingler et al., 2012). In this study, high S induced decreases in Tre and Tre6P concentrations. Simultaneously, ABA, which is an indicator of plant senescence, was decreased with high S at both N levels. The results suggested that carbohydrate metabolism and leaf senescence-related signal pathways were involved in S-promoted AGC biosynthesis in *A. paniculata*.

GAs, SA, and Tre6P link plant development and metabolism. Although Tre6P and phytohormones are interactive in the regulation of some of those processes (O’Hara et al., 2013; Meitzel et al., 2020), the crosstalk of GAs, SA, and Tre6P in the regulation of AGC biosynthesis remains to be elucidated. Furthermore, it is worth noting that the signaling pathways involved in S-mediated AGC biosynthesis were similar at different N levels, but the growth and metabolism varied greatly in response to S. It is implied that S has relatively independent regulatory mechanisms on the growth and secondary metabolism in *A. paniculata*.

## Conclusion

The results revealed that enhancing the S application rate promotes the biosynthesis of AGCs in *A. paniculata* plants, and that the interaction of N and S influences the trade-off between plant growth and AGC accumulation. N metabolism plays a key role in this process. High S promoted N assimilation and S metabolism with increases in the TCA cycle and photorespiration under low N conditions, leading to increased consumption of carbohydrates and inhibited growth. In contrast, under high N conditions, increasing S application reduced N and S accumulation, and promoted the coordination between plant growth and AGC accumulation. SA, GA_4_, and Tre6P showed a similar response to S at both N levels and were indicated to be involved in S-mediated AGC accumulation in *A. paniculata* plants. These results suggested that the regulatory mechanisms of S on growth and secondary metabolism are relatively independent. However, further studies are needed to reveal the crosstalk between SA, GA_4_, and Tre6P in the regulation of andrographolide biosynthesis, and the relationship between N metabolism and secondary metabolism.

## Data Availability Statement

The original contributions presented in the study are included in the article/Supplementary Material, further inquiries can be directed to the corresponding authors.

## Author Contributions

CZ and J-HM designed the experiments. S-FJ drafted the manuscript, and CZ revised it. S-FJ, X-JH, and X-NY carried out the experiments and analyzed the samples and data. All authors contributed to the article and approved the submitted version.

### Conflict of Interest

The authors declare that the research was conducted in the absence of any commercial or financial relationships that could be construed as a potential conflict of interest.

## Figures and Tables

**Table 1:** 

Term	Definitions
ABA	Abscisic acid
AGCs	Andrographolide compounds
CNBH	Carbon/nutrient balance hypothesis
Cys	Cysteine
E4P	Erythrose 4-phosphate
GA	Gibberellic acid
GS	Glutamine synthetase
GOT	Glutamic-oxaloacetic transaminase
GPT	Glutamic-pyruvic transaminase
JA	Jasmonate
MEP	Methylerythritol phosphate
MeJA	Methyl jasmonate
Met	Methionine
MVA	Mevalonic acid
N	Nitrogen
NR	Nitrate reductase
PEP	Phosphoenolpyruvate
PPP	Pentose phosphate pathway
S	Sulfur
SA	Salicylic acid
SAH	S-Adenosyl-l-homocysteine
SAM	S-Adenosyl-methionine
Tre	Trehalose
Tre6P	Trehalose 6-phosphate

## Footnotes

^1^https://www.mzcloud.org/

^2^http://www.chemspider.com/
