# Supplementary material for: Sulfur Regulates the Trade-Off Between Growth and Andrographolide Accumulation via Nitrogen Metabolism in Andrographis paniculata
Source: Front Plant Sci. 2021 Jul 14;12:687954. doi: 10.3389/fpls.2021.687954 (PMC8317024; doi:10.3389/fpls.2021.687954)
Supplement: Supplementary file 1 [file Data_Sheet_1.docx]

**Table S1** Chemical composition of nutrient solution used in this study

| Compounds | Unit | N_4_S_0.1_ | N_4_S_2.4_ | N_8_S_0.1_ | N_8_S_2.4_ |
| --- | --- | --- | --- | --- | --- |
| KH_2_PO_4_ | mmol·L^-1^ | 0.4 | 0.4 | 0.4 | 0.4 |
| KCl | mmol·L^-1^ | 1.8 | -- | 1.8 | -- |
| K_2_SO_4_ | mmol·L^-1^ | -- | 0.9 | -- | 0.9 |
| MgSO_4_ | mmol·L^-1^ | 0.1 | 0.5 | 0.1 | 0.5 |
| MgCl_2_ | mmol·L^-1^ | 0.4 | -- | 0.4 | -- |
| CaCl_2_ | mmol·L^-1^ | 2.5 | 1.5 | 2.5 | 1.5 |
| Ca(NO_3_)_2_ | mmol·L^-1^ | -- | 1.0 | -- | 1.0 |
| (NH_4_)_2_SO_4_ | mmol·L^-1^ | -- | 1.0 | -- | 1.0 |
| NH_4_NO_3_ | mmol·L^-1^ | 2.0 | -- | 4.0 | 2.0 |
| H_3_BO_3_ | μmol·L^-1^ | 18.0 | 18.0 | 18.0 | 18.0 |
| (NH_4_)_6_Mo_7_O_24_ | μmol·L^-1^ | 0.1 | 0.1 | 0.1 | 0.1 |
| CuSO_4_ | μmol·L^-1^ | 0.15 | 0.15 | 0.15 | 0.15 |
| ZnCl_2_ | μmol·L^-1^ | 0.15 | 0.15 | 0.15 | 0.15 |
| MnCl_2_ | μmol·L^-1^ | 3.5 | 3.5 | 3.5 | 3.5 |
| Fe-EDTA | μmol·L^-1^ | 36.0 | 36.0 | 36.0 | 36.0 |


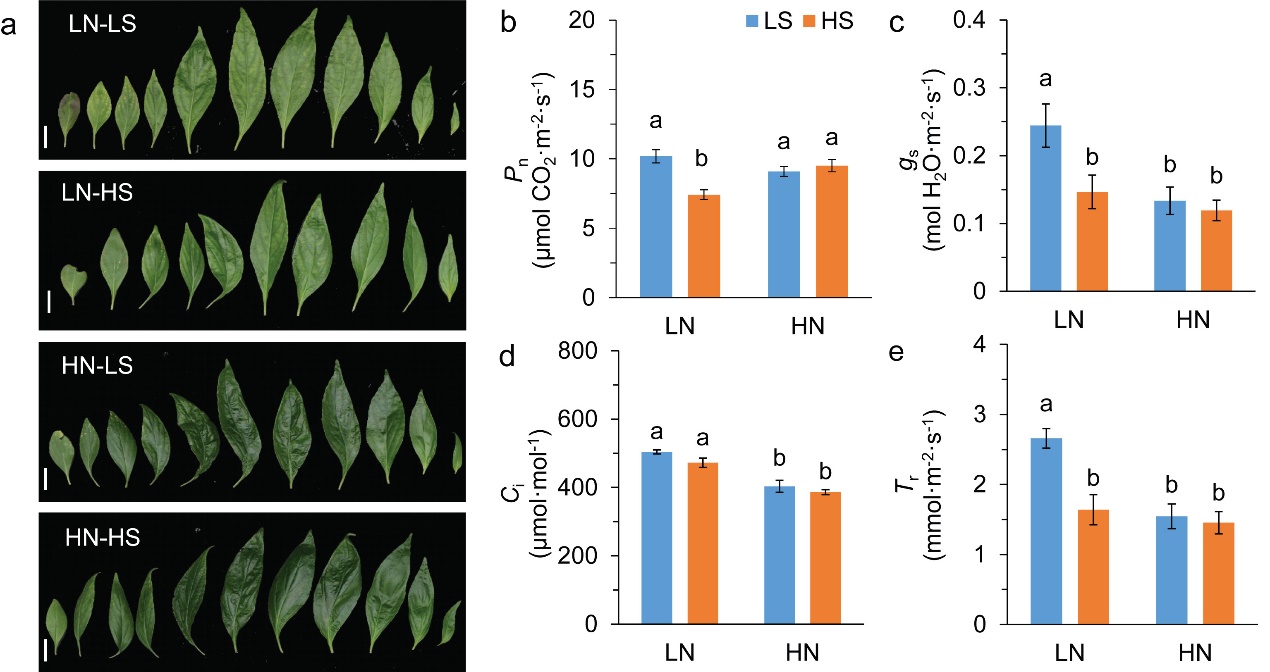


**Fig.S1** Phenotype of leaf position and leaf gas exchange parameters. **a**, leaf position in main stem; **b**, net photosynthetic rate; **c**, stomatal conductance; **d**, intercellular CO_2_ concentration; **e**, transpiration rate. Data were presented as mean ± SE (*n* = 4). Different letters on the bars indicates significant difference among treatments at *P* < 0.05 using the method of *LSD*. Scar bar=1 cm in **a**. LN, low nitrogen; HN, high N; LS, low sulfur; HS, high sulfur.


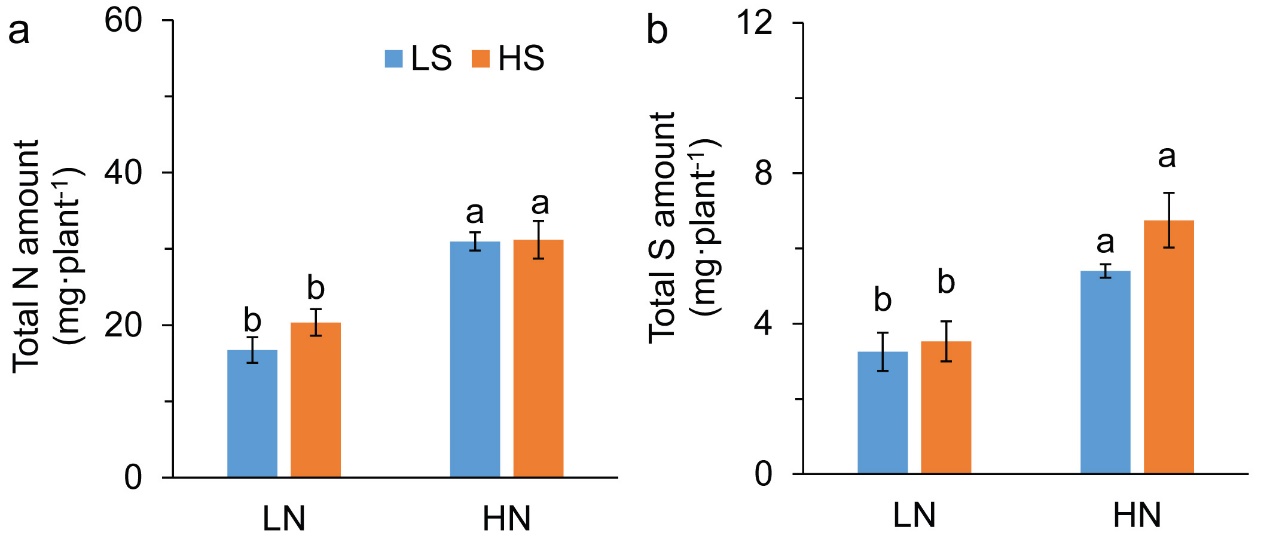


**Fig.S2** Total amounts of leaf nitrogen (**a**) and sulfur (**b**) based on per plant. Data were presented as mean ± SE (*n* = 4). Different letters on the bars indicates significant difference among treatments at *P* < 0.05 using the method of *LSD*. LN, low nitrogen; HN, high N; LS, low sulfur; HS, high sulfur.


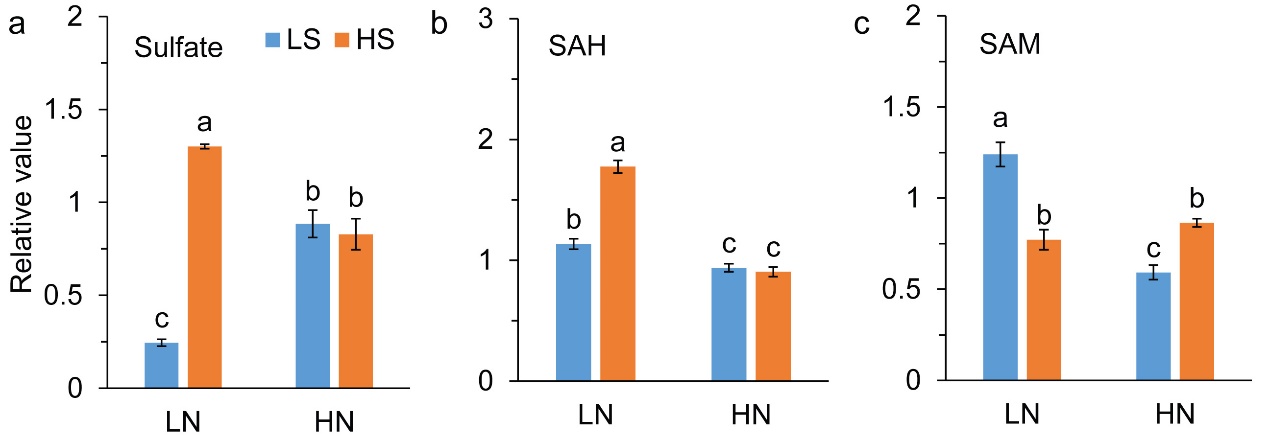


**Fig.S3** Contents of sulfate (**a**), S-adenosyl-L-homocysteine (**b**), and S-adenosyl-methionine (**c**). Data were presented as mean ± SE (*n* = 4). Different letters on the bars indicates significant difference among treatments at *P* < 0.05 using the method of *LSD*. LN, low nitrogen; HN, high N; LS, low sulfur; HS, high sulfur.
